# Omega-3 Long-Chain Polyunsaturated Fatty Acid Content and Oxidation State of Fish Oil Supplements in New Zealand

**DOI:** 10.1038/s41598-017-01470-4

**Published:** 2017-05-03

**Authors:** Gerard Bannenberg, Craig Mallon, Holly Edwards, Derek Yeadon, Kevin Yan, Holly Johnson, Adam Ismail

**Affiliations:** 1 Global Organization for EPA and DHA omega-3s (GOED), 1075 Hollywood Ave, Salt Lake City, UT 84105 USA; 2 DSM Nutritional Products, 6480 Dobbin Road, Columbia, MD 21045 USA; 3 Omega Protein, 6961 Brookhollow West Dr., Suite 190, Houston, TX 77040-3256 USA; 4 Eurofins Central Analytical Laboratories, 2219 Lakeshore Drive, Suite 100, New Orleans, LA 70122 USA; 5 Nutrasource Diagnostics Inc, 203-120 Research lane, Guelph, ON N1G 0B4 Canada; 6 Alkemist Labs, 1260 Logan Ave, Costa Mesa, CA 92626 USA

## Abstract

Forty-seven fish oil products available on the New Zealand market were analyzed for eicosapentaenoic acid (EPA) and docosahexaenoic acid (DHA) content, as well as for oxidative status in a collaborative effort by several analytical laboratories. Of the tested products, 72%, 86% and 77% complied with voluntary industry-set maximum limits on Peroxide Value (PV), *para*-Anisidine Value (p-AV), and TOTOX, respectively. 91% of the products complied with EPA/DHA content claims. All fish oils complied with a p-AV limit of 30, 98% with a PV limit of 10 meq O_2_/kg, and 96% with a calculated TOTOX value of 50, which are less stringent limits used by the European and British Pharmacopeia and the Australian authorities. The results are in stark contrast to the very low percentage of fish oil products reported to be in compliance with primary oxidation limits and EPA/DHA content by a recently published assessment of fish oil supplements in New Zealand. Possible reasons for this discrepancy are evaluated and discussed.

## Introduction

Omega-3 long-chain polyunsaturated fatty acids (LCPUFA) are substances with a range of important structural and regulatory functions in human and animal physiology. Deficiencies in their dietary intake and tissue levels are related to chronic disease susceptibility^1^. Dietary supplementation with preformed EPA and DHA is a convenient way to complement a diet that is low in omega-3 LCPUFA. A range of dietary supplements rich in EPA/DHA is available to consumers. A substantial number of these finished products are produced with EPA/DHA-containing oils derived from marine sources, notably from anchovy and cod liver. Other sources, such as purposefully grown microalgae, are of growing importance to address global omega-3 LCPUFA intake deficiencies.

Polyunsaturated fatty acids are biosynthesized and used by many organisms, and serve as dedicated substrates to be enzymatically oxidized in a stereochemically- and positionally-controlled manner, *e.g*. in the generation of intercellular signaling molecules. The polyunsaturated nature that allows their oxygenation also renders these substances highly sensitive to oxidation by molecular oxygen (O_2_) present in air when taken out of the biological context^2^. A fundamental difference between enzymatic and non-enzymatic oxidation reactions of PUFA resides in the capability of fatty acid oxygenases to control the formation of fatty acid-peroxyl radicals. In the biosynthesis of fatty acid peroxides as intermediates in autacoid formation, the peroxyl radical that is formed upon reaction of a PUFA with molecular oxygen, is immediately reduced within the enzyme active site to form the corresponding peroxide. However, in non-enzymatic oxidation reactions involving PUFA, the production of a fatty acid peroxide involves the transient formation of a peroxyl radical that is free to abstract a hydrogen atom from any nearby hydrogen-donating substance. This can be an antioxidant, for example tocopherol, or when the concentrations of reduced anti-oxidants are too low to efficiently scavenge peroxyl radicals, any closely-situated PUFA molecule. Free radical-mediated propagation of lipid peroxidation can lead to rapid deterioration of a PUFA-rich oil exposed to oxygen. Even in the presence of hydrogen-donating antioxidants, fatty acid peroxides will still be formed during chemical oxidation of PUFA, but free radical-mediated propagation is abrogated.

As chemical lipid peroxidation advances, secondary reactions involving the formed fatty acid peroxides will also take place. Of particular importance to neat oils, alkoxy radicals derived from the initially formed peroxides can undergo addition reactions with conjugated double bonds (formed as a result of earlier hydrogen abstraction-promoted double bond rearrangements) and favor the cleavage of the fatty acid chain with corresponding formation of chain-shortened α, β-unsaturated aldehydes^3^. The production of secondary oxidation products can be viewed as a “later” stage of non-enzymatic lipid peroxidation that depends on the initial formation and ensuing consumption of fatty acid peroxides. The detailed outcome of free-radical-mediated oxidation reactions involving PUFA-containing systems is exceedingly complex and depends on many factors^4^. Under ambient and real-life conditions, the absolute oxidative stability of EPA/DHA-containing oils cannot be achieved, but it can be slowed down considerably by limiting the exposure to air, and by reducing the rates of peroxidation and additional reactions secondary to primary oxidation, for example by maintaining sufficiently high concentrations of reduced antioxidants, and limiting exposure to predisposing factors such as heat and light.

The quality of EPA- and DHA-containing oils used in dietary supplements, as well as pharmaceutical ingredients, is specified in pharmacopeial monographs, as well as by regulations with regional competence. Quality usually encompasses the oxidation state, fatty acid content, and presence of environmental contaminants, as well as the methods employed to measure these in various classes of marine oils. The organization responsible for the present report, GOED (Global Organization for EPA and DHA Omega-3s), is an association of producers of omega-3 LCPUFA-containing products, as well as associated industry activities. Members of GOED are required to produce omega-3 LCPUFA-rich oils that comply with certain limits on primary oxidation (Peroxide Value; PV < 5 meq O_2_/kg), secondary oxidation (*para*-Anisidine Value; p-AV < 20), and a combined measurement of total oxidation that takes both the level of primary and secondary oxidation into account (TOTOX < 26)^5^. These requirements are only applicable to products within the scope of the GOED Monograph, and members are not required to follow these guidelines for products outside the scope. The limits are at least as strict or stricter than many pharmacopeial monographs and global regulations. The rest of the omega-3 oil industry is encouraged to follow GOED guidelines. The limits are also below oxidation levels seen in other ingredients or food items available to consumers, as well as the maximum limits for oxidation for seed and plant oils that are also sensitive to oxidation^6, 7^. In addition to monitoring ingredient and product quality, the accurate measurement of fatty acid content and oxidative status is fundamental to permitting the proper assessment of the biological relevance of different types of omega-3 LCPUFA-containing oils, as well as the dependence of the biological effect on the concentration and type of any oxidation products that may be present in oils, in combination with dose- and time-dependent exposure^8–10^.

Precautions during sample handling and preparation, the use of adequate analytical separation and detection techniques, and proper expression of results are important to obtaining correct results in quality assessments of PUFA-rich oil products. Several critical aspects to obtaining correct quantitative polyunsaturated fatty acid measurements and avoiding inadvertent oxidation include: (i) do not store pooled oils isolated from capsules (or any formulation), but process each individual oil sample immediately (within minutes) for analysis, (ii) ascertain that the laboratory materials used for oil sample containment and transfer during sample preparation do not promote oxidation (*e.g*. use low-actinic glassware, and be careful with the use of common plastic laboratory disposable tubes and improperly washed glass tubes that can be a source of transition metal contamination), (iii) work under nitrogen or argon whenever possible (for example during sample evaporation steps, and sparging of solvents to lower oxygen tension), (iv) ascertain sufficient presence of reduced antioxidant(s), (v) use liners that can accommodate the injected sample volume in gas chromatographic analyses, (vi) use both internal and external standards in quantitative fatty acid analysis, (vii) obtain baseline resolution, correct peak assignment, and confirm integration of gas chromatographic signals belonging to specific fatty acids, and (viii) pay close attention to system suitability acceptance criteria. The participation in laboratory proficiency programs (*e.g*. those organized by the American Oil Chemistry Society^11^ or the National Institutes of Standards and Technology^12^) and accreditation schemes is furthermore recommended for any laboratory to assess analytical proficiency.

Several studies have been published in recent years that have assessed the oxidative quality and fatty acid content of encapsulated and liquid formulations of EPA/DHA. Whereas some studies concluded that most tested products were compliant^13–16^, other studies have reported a significant proportion of tested products failing one or more quality parameters^17–27^. In particular, one study published in this journal reported that 69% of 32 analyzed fish oil products available to consumers in New Zealand^28^ contained less EPA/DHA than the product label indicated, along with a significant proportion of the products surpassing maximum oxidation limits used by GOED. Of the analyzed products, 83% were reported to exceed the limit on PV, 25% on p-AV, and 50% on TOTOX. Based on the observations, the study reported a relationship between the level of oxidation and “missing” EPA/DHA, and suggested that oxidation could contribute to the decrease in EPA/DHA content.

The reported elevated rate of fish oil product non-compliance does not reflect the results of randomized market surveillance testing of finished products carried out by GOED since 2009. It also does not align with reports published by third-party product quality monitors, including Consumer Reports^29^, Labdoor.com ^30^, ConsumerLab.com ^31^, and the International Fish Oil Standards Program (IFOS)^32^. The divergent results encouraged the undertaking of the present investigation that aimed to verify the results from Albert and colleagues^28^, and if divergent to identify possible sources of error that may have caused a discrepancy and report a more realistic expected level of compliance of fish oil products sold in New Zealand.

## Results

The current study evaluated the oxidative quality and EPA/DHA content of 47 fish oil products available in New Zealand.

### Oxidative status

Results from the independent analyses by two different laboratories of the oxidative status of each sample are provided in Table 1 (PV), Table 2 (p-AV) and Table 3 (TOTOX). The results show only minor interlaboratory differences for PV on all oils, and for p-AV and TOTOX on the unflavored oils to which the limits are applicable. Average values for each of the parameters for each product were calculated when more than a single laboratory provided results. Of the fish oil products tested for PV, 13 (28%) exceeded 5 meq O_2_/kg (Table 1). Four products could only be tested for PV by one laboratory due to insufficient sample material and/or matrix interference. Three of the 22 unflavored fish oil products (14%) exceeded the p-AV limit (Table 2). Based on calculated TOTOX values of unflavored fish oil products, five of the 22 exceeded the TOTOX limit (23%) (Table 3).

**Table 1:** Peroxide Value test results (meq O_2_/kg).

Company	Brand	Product Name	Laboratory			
			Eurofins	Nutrasource	Omega Protein	Average
Sanofi Consumer Healthcare	Nature’s Own	Fish Oil 1000 mg	8.34		9.81	9.08*
Sanofi Consumer Healthcare	Nature’s Own	Fish Oil 2000 mg	7.98		9.02	8.50*
Sanofi Consumer Healthcare	Nature’s Own	Odourless Fish Oil 1500 mg	4.86	5.83		5.35*
Sanofi Consumer Healthcare	Nature’s Own	Odourless Fish Oil 2000mg	3.40	4.06		3.73
Sanofi Consumer Healthcare	Nature’s Own	Odourless Fish Oil 1000 mg	5.57	6.59		6.08*
Sanofi Consumer Healthcare	Nature’s Own	Concentrated Fish Oil 1-A-Day Odourless	0.99			0.99
Swisse Wellness	Swisse	Ultiboost Odourless Wild Fish Oil	6.37	7.39		6.88*
Good Health Products	Good Health	Omega 3 Fish Oil 1500 mg	2.47		3.92	3.20
Good Health Products	Good Health	Omega 3 Fish Oil Health Guard 1000 mg	3.30		4.13	3.72
Good Health Products	Good Health	Super Omega 3 Health Guard Fish Oil	1.05		1.96	1.51
Good Health Products	Good Health	O-Mega 3 Bursts	4.16	3.29		3.73
Integria Healthcare	Thompson’s	Fish Oil 1500	3.21	3.22		3.22
Integria Healthcare	Thompson’s	Salmon Oil Plus 1000 mg	0.71		1.26	0.99
Vitaco Health	NutraLife	Fish Oil 1000 mg	1.69		2.20	1.95
Vitaco Health	NutraLife	OceanClean Triple Strength Omega 3	2.36	2.16		2.22
Vitaco Health	NutraLife	Fish Oil 1500 mg	3.32		4.47	3.90
Vitaco Health	NutraLife	Fish Oil 1500 mg Plus Vitamin D	1.89		3.30	2.60
Vitaco Health	NutraLife	Omega Smart Bites	3.86	4.55		4.21
Real Vitamins	Sanderson	Fish Oil 2000	2.38	2.97		2.68
Real Vitamins	Sanderson	Fish Oil 3000	4.31	5.00		4.66
Comvita New Zealand	Comvita	Omega 3 Fish Oil	2.38		3.00	2.69
Nordic Naturals	Nordic Naturals	Ultimate Omega	3.75	3.87		3.81
Health & Herbs International	Radiance	Omega 3 EPA & DHA	3.16		3.90	3.53
Red Seal Natural Health	Red Seal	High Potency Fish Oil 1,500 mg	2.99		4.12	3.56
Lighthouse Supplements	Lighthouse	Salmon Oil 1000	1.99		2.37	2.18
Vitaco Health	Healtheries	Fish Oil 1000 mg	3.18		4.32	3.75
Vitaco Health	Healtheries	Fish Oil 1500 mg	2.96		4.09	3.53
BioBalance New Zealand	BioBalance	Omega 3 Fish Oil	1.58		2.00	1.79
Natural Health Laboratories	Clinicians	Omega-3 Fish Oil 1000 mg	6.36	7.55		6.96*
Natural Health Laboratories	Clinicians	Omega-3 Fish Oil 1500 mg High Strength	4.23	5.31		4.77
Health World	Ethical Nutrients	Hi-Strength Fish Oil Capsules	5.23	5.58		5.41*
Sanofi Consumer Healthcare	MicroGenics	1500 mg Odourless Fish Oil	5.53	6.38		5.96*
Sanofi Consumer Healthcare	MicroGenics	Wild 1000 mg Fish Oil	8.61		9.75	9.18*
GO Healthy New Zealand	GO Healthy	GO Fish Oil Reflux Free 1,000 mg	3.44	4.31		3.88
GO Healthy New Zealand	GO Healthy	GO Fish Oil 1-A-Day + Vitamin D3 1,000IU	4.76		5.64	5.20*
GO Healthy New Zealand	GO Healthy	GO Fish Oil 2,000 mg	6.60	7.26		6.93*
GO Healthy New Zealand	GO Healthy	GO Fish Oil 1,000 mg Odourless	1.33		1.90	1.62
GO Healthy New Zealand	GO Healthy	GO Fish Oil 1,500 mg Odourless	3.29		4.53	3.91
PharmaCare Laboratories	Bioglan	Kids Smart Omega-3 Fish Oil	1.91	2.00		1.96
PharmaCare Laboratories	Bioglan	Super Fish Oil	18.30		11.45	14.88*
PharmaCare Laboratories	Bioglan	Kids Smart Omega-3 Fish Oil Trio	2.45	2.00		2.23
Blackmores	Blackmores	Fish Oil 1000*	4.58		5.07	4.83
Blackmores	Blackmores	Odourless Fish Oil 1000	3.18	3.85		3.52
Blackmores	Blackmores	Omega Triple	3.97	4.41		4.19
Blackmores	Blackmores	Omega Daily	2.50	2.98		2.74
Blackmores	Blackmores	Omega Joint		2.22		2.22
Blackmores	Blackmores	Omega Brain		5.02		5.02*

^*^Indicates products where the average result from all laboratories exceeded the GOED Voluntary Monograph limit of 5 meq O_2_/kg.

**Table 2:** p-Anisidine Value test results.

Company	Brand	Product Name	Laboratory			
			Eurofins	Nutrasource	Omega Protein	Average
**Unflavored Oils**						
Sanofi Consumer Healthcare	Nature’s Own	Fish Oil 1000 mg	12.40		15.96	14.18
Sanofi Consumer Healthcare	Nature’s Own	Fish Oil 2000mg	13.40		16.89	15.15
Good Health Products	Good Health	Omega 3 Fish Oil 1500 mg	10.80		12.61	11.71
Good Health Products	Good Health	Omega 3 Fish Oil Health Guard 1000 mg	9.90		12.04	10.97
Good Health Products	Good Health	Super Omega 3 Health Guard Fish Oil	3.60		5.74	4.67
Integria Healthcare	Thompson’s	Salmon Oil Plus 1000 mg	24.10		24.27	24.19*
Vitaco Health	NutraLife	Fish Oil 1000 mg	11.30		12.31	11.81
Vitaco Health	NutraLife	Fish Oil 1500 mg	11.60		12.28	11.94
Vitaco Health	NutraLife	Fish Oil 1500 mg Plus Vitamin D	11.10		12.23	11.67
Comvita New Zealand	Comvita	Omega 3 Fish Oil	13.10		13.16	13.13
Health & Herbs International	Radiance	Omega 3 EPA & DHA	10.50		12.32	11.41
Red Seal Natural Health	Red Seal	High Potency Fish Oil 1,500 mg	11.50		12.84	12.17
Lighthouse Supplements	Lighthouse	Salmon Oil 1000	19.60		22.81	21.21^*^
Vitaco Health	Healtheries	Fish Oil 1000 mg	15.90		11.49	13.70
Vitaco Health	Healtheries	Fish Oil 1500 mg	13.40		11.98	12.69
BioBalance New Zealand	BioBalance	Omega 3 Fish Oil	16.00		12.19	14.10
Sanofi Consumer Healthcare	MicroGenics	Wild 1000 mg Fish Oil	11.40		15.52	13.46
GO Healthy New Zealand	GO Healthy	GO Fish Oil 1-A-Day + Vitamin D3 1,000IU	3.30		9.36	6.33
GO Healthy New Zealand	GO Healthy	GO Fish Oil 1,000 mg Odourless	5.30		11.83	8.57
GO Healthy New Zealand	GO Healthy	GO Fish Oil 1,500 mg Odourless	4.30		12.32	8.31
PharmaCare Laboratories	Bioglan	Super Fish Oil	27.60		22.25	24.93*
Blackmores	Blackmores	Fish Oil 1000*	9.20		12.59	10.90
**Flavored Oils**						
Sanofi Consumer Healthcare	Nature’s Own	Odourless Fish Oil 1500 mg	19.60	16.53		18.07
Sanofi Consumer Healthcare	Nature’s Own	Odourless Fish Oil 2000mg	18.10	15.33		16.72
Sanofi Consumer Healthcare	Nature’s Own	Odourless Fish Oil 1000 mg	20.00	27.72		23.86*
Sanofi Consumer Healthcare	Nature’s Own	Concentrated Fish Oil 1-A-Day Odourless	22.40	**		22.40*
Swisse Wellness	Swisse	Ultiboost Odourless Wild Fish Oil	27.10	25.29		26.20*
Good Health Products	Good Health	O-Mega 3 Bursts	40.80	36.70		38.75*
Integria Healthcare	Thompson’s	Fish Oil 1500	84.30	40.03		62.17*
Vitaco Health	NutraLife	OceanClean Triple Strength Omega 3	162.80	31.46		75.24*
Vitaco Health	NutraLife	Omega Smart Bites	47.80	26.59		37.20*
Real Vitamins	Sanderson	Fish Oil 2000	22.00	17.11		19.56
Real Vitamins	Sanderson	Fish Oil 3000	17.80	9.83		13.82
Nordic Naturals	Nordic Naturals	Ultimate Omega	43.60	30.27		36.94*
Natural Health Laboratories	Clinicians	Omega-3 Fish Oil 1000 mg	90.80	32.42		61.61*
Natural Health Laboratories	Clinicians	Omega-3 Fish Oil 1500 mg High Strength	92.50	16.47		54.49*
Health World	Ethical Nutrients	Hi-Strength Fish Oil Capsules	54.70	30.59		42.65*
Sanofi Consumer Healthcare	MicroGenics	1500 mg Odourless Fish Oil	17.70	15.98		16.84
GO Healthy New Zealand	GO Healthy	GO Fish Oil Reflux Free 1,000 mg	5.60	10.23		7.92
GO Healthy New Zealand	GO Healthy	GO Fish Oil 2,000 mg	4.70	11.29		8.00
PharmaCare Laboratories	Bioglan	Kids Smart Omega-3 Fish Oil	**	4.46		4.46
PharmaCare Laboratories	Bioglan	Kids Smart Omega-3 Fish Oil Trio	**	6.89		6.89
Blackmores	Blackmores	Odourless Fish Oil 1000	65.10	41.27		53.19*
Blackmores	Blackmores	Omega Triple	29.40	20.45		24.93*
Blackmores	Blackmores	Omega Daily	32.30	33.79		33.05*
Blackmores	Blackmores	Omega Joint	***	29.28		29.28*
Blackmores	Blackmores	Omega Brain	***	19.80		19.80

^*^Indicates products where the average result from all laboratories exceeded the GOED Voluntary Monograph limit for p-AV of 20. In the flavored oils these ar*e* noted for purposes of illustration only, as the p-AV limit is not applicable to flavored oils due to the known interference of flavorings with the method.

^**^Indicates products for which insufficient sample material was available for testing.

^***^Indicates products for which insufficient funding for tests was available.

**Table 3:** TOTOX test results.

Company	Brand	Product Name	Laboratory			
			Eurofins	Nutrasource	Omega Protein	Average
**Unflavored Oils**						
BioBalance New Zealand	BioBalance	Omega 3 Fish Oil	19.16		16.19	17.68
Blackmores	Blackmores	Fish Oil 1000*	18.36		22.73	20.55
Comvita New Zealand	Comvita	Omega 3 Fish Oil	17.86		19.16	18.51
GO Healthy New Zealand	GO Healthy	GO Fish Oil 1,000 mg Odourless	7.96		15.63	11.80
GO Healthy New Zealand	GO Healthy	GO Fish Oil 1,500 mg Odourless	10.88		21.38	16.13
GO Healthy New Zealand	GO Healthy	GO Fish Oil 1-A-Day + Vitamin D3 1,000IU	12.82		20.64	16.73
Good Health Products	Good Health	Omega 3 Fish Oil 1500 mg	15.74		20.45	18.10
Good Health Products	Good Health	Omega 3 Fish Oil Health Guard 1000 mg	16.5		20.3	18.40
Good Health Products	Good Health	Super Omega 3 Health Guard Fish Oil	5.7		9.66	7.68
Health & Herbs International	Radiance	Omega 3 EPA & DHA	16.82		20.12	18.47
Integria Healthcare	Thompson’s	Salmon Oil Plus 1000 mg	25.52		26.79	26.16*
Lighthouse Supplements	Lighthouse	Salmon Oil 1000	23.58		27.55	25.57
PharmaCare Laboratories	Bioglan	Super Fish Oil	64.2		45.15	54.68*
Red Seal Natural Health	Red Seal	High Potency Fish Oil 1,500 mg	17.48		21.08	19.28
Sanofi Consumer Healthcare	MicroGenics	Wild 1000 mg Fish Oil	28.62		35.02	31.82*
Sanofi Consumer Healthcare	Nature’s Own	Fish Oil 1000 mg	29.08		35.58	32.33*
Sanofi Consumer Healthcare	Nature’s Own	Fish Oil 2000mg	29.36		34.93	32.15*
Vitaco Health	Healtheries	Fish Oil 1000 mg	22.26		20.13	21.20
Vitaco Health	Healtheries	Fish Oil 1500 mg	19.32		20.16	19.74
Vitaco Health	NutraLife	Fish Oil 1000 mg	14.68		16.71	15.70
Vitaco Health	NutraLife	Fish Oil 1500 mg	18.24		21.22	19.73
Vitaco Health	NutraLife	Fish Oil 1500 mg Plus Vitamin D	14.88		18.83	16.86
**Flavored Oils**						
Blackmores	Blackmores	Odourless Fish Oil 1000	71.46	48.97		60.22*
Blackmores	Blackmores	Omega Brain	***	29.84		29.84*
Blackmores	Blackmores	Omega Daily	37.3	39.75		38.53*
Blackmores	Blackmores	Omega Joint	***	33.72		33.72*
Blackmores	Blackmores	Omega Triple	37.34	29.27		33.31*
GO Healthy New Zealand	GO Healthy	GO Fish Oil 2,000 mg	17.9	25.81		21.86
GO Healthy New Zealand	GO Healthy	GO Fish Oil Reflux Free 1,000 mg	12.48	18.85		15.67
Good Health Products	Good Health	O-Mega 3 Bursts	49.12	43.28		46.20*
Health World	Ethical Nutrients	Hi-Strength Fish Oil Capsules	65.16	41.75		53.46*
Integria Healthcare	Thompson’s	Fish Oil 1500	90.72	46.47		68.60*
Natural Health Laboratories	Clinicians	Omega-3 Fish Oil 1000 mg	103.52	47.52		75.52*
Natural Health Laboratories	Clinicians	Omega-3 Fish Oil 1500 mg High Strength	100.96	27.09		64.03*
Nordic Naturals	Nordic Naturals	Ultimate Omega	51.1	38.01		44.56*
PharmaCare Laboratories	Bioglan	Kids Smart Omega-3 Fish Oil	**	8.46		8.46
PharmaCare Laboratories	Bioglan	Kids Smart Omega-3 Fish Oil Trio	**	10.89		10.89
Real Vitamins	Sanderson	Fish Oil 2000	26.76	23.05		24.91
Real Vitamins	Sanderson	Fish Oil 3000	26.42	19.83		23.13
Sanofi Consumer Healthcare	MicroGenics	1500 mg Odourless Fish Oil	28.76	28.74		28.75*
Sanofi Consumer Healthcare	Nature’s Own	Concentrated Fish Oil 1-A-Day Odourless	24.38	**		24.38
Sanofi Consumer Healthcare	Nature’s Own	Odourless Fish Oil 1000 mg	31.14	40.9		36.02*
Sanofi Consumer Healthcare	Nature’s Own	Odourless Fish Oil 1500 mg	29.32	28.19		28.76*
Sanofi Consumer Healthcare	Nature’s Own	Odourless Fish Oil 2000 mg	24.9	23.45		24.18
Swisse Wellness	Swisse	Ultiboost Odourless Wild Fish Oil	39.84	40.07		39.96*
Vitaco Health	NutraLife	OceanClean Triple Strength Omega 3	167.52	35.77		79.69*
Vitaco Health	NutraLife	Omega Smart Bites	55.52	35.69		45.61*

^*^Indicates products where the average result from all laboratories exceeded the GOED Voluntary Monograph limit for TOTOX of 26. In the flavored oils these are noted for purposes of illustration only, as the TOTOX limit is not applicable to flavored oils due to the known interference of flavorings with the p-AV portion of the TOTOX calculation.

^**^Indicates products for which insufficient sample material was available for testing.

**Figure 1:**
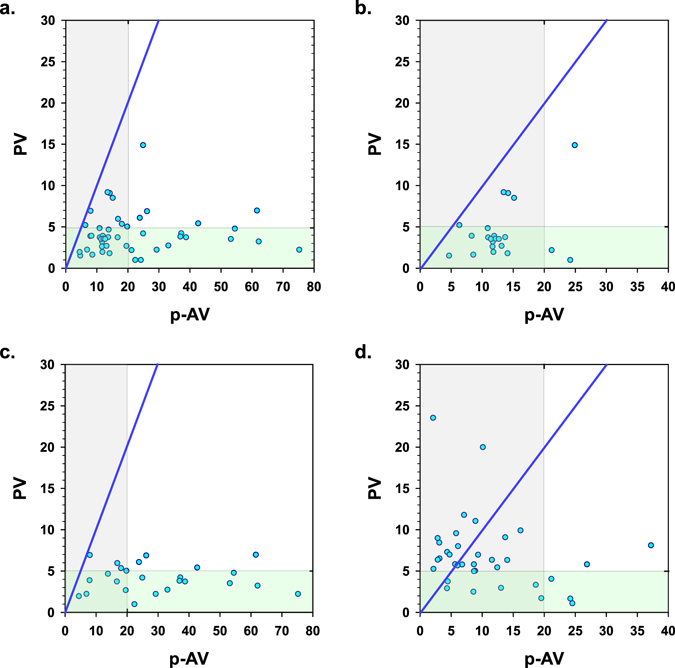
Comparison of the ratio of PV to p-AV of New Zealand fish oil products tested in the present study (panel a: all tested products, panel b: only unflavored oils, panel c: only flavored oils) with those reported by Albert *et al*.^28^ (panel d). The green- and grey-shaded areas fall below the GOED maximum PV and p-AV limits of 5 meq O_2_/kg and 20, respectively. The blue line represents a PV/p-AV ratio of 1.

Of the 21 flavored fish oil products that were tested using the TAV test, a specialized method allowing secondary oxidation to be measured in the presence of flavors (see Methods), 4 products were found to have secondary oxidation levels that were considered too high (data not shown). Five flavored products could not be analyzed using this methodology. For comparison, 15 of 25 flavored products had an average p-AV > 20 (60%) and 17 of 25 products had an average TOTOX > 26 (68%), although these limits are not applicable to oils with added flavors. These results are also displayed graphically in Fig. 1a (all products), Fig. 1b (all unflavored fish oils), and Fig. 1c (all flavored fish oils).

### EPA/DHA content

Results of the analysis of EPA plus DHA content per capsule (mg fatty acid expressed as triglycerides) of each product by two or three different laboratories are provided in Table 4. The average value of the independent analyses was calculated and compared with the content claimed on the product label, and expressed as % of label claim. Four of the 47 fish oil products (8.5%) had an EPA/DHA content below 90% of the content claim.

**Table 4:** EPA + DHA content of fish oil products (mg EPA plus DHA per capsule).

Company	Brand	Product Name	Alkemist	DSM	Eurofins	Nutrasource	Omega Protein	Average	Label Claim	% of Label Claim
BioBalance New Zealand	BioBalance	Omega 3 Fish Oil			285		260	273	300	91%
Blackmores	Blackmores	Fish Oil 1000*			289		296	292	300	97%
Blackmores	Blackmores	Odourless Fish Oil 1000		290	288	264		281	300	94%
Blackmores	Blackmores	Omega Brain	636			585		610	600	102%
Blackmores	Blackmores	Omega Daily	611		559	528		566	600	94%
Blackmores	Blackmores	Omega Joint		747		698		722	670	108%
Blackmores	Blackmores	Omega Triple		851	844	784		826	900	92%
Comvita New Zealand	Comvita	Omega 3 Fish Oil			286		269	278	300	93%
GO Healthy New Zealand	GO Healthy	GO Fish Oil 1,000mg Odourless			280		257	269	300	90%
GO Healthy New Zealand	GO Healthy	GO Fish Oil 1,500 mg Odourless			440		436	438	450	97%
GO Healthy New Zealand	GO Healthy	GO Fish Oil 1-A-Day + Vitamin D3 1,000IU			751		716	734	750	98%
GO Healthy New Zealand	GO Healthy	GO Fish Oil 2,000 mg		507	500	475		494	600	82%*
GO Healthy New Zealand	GO Healthy	GO Fish Oil Reflux Free 1,000 mg	299		291	266		285	300	95%
Good Health Products	Good Health	O-Mega 3 Bursts	241		222	205		222	233	95%
Good Health Products	Good Health	Omega 3 Fish Oil 1500 mg			429		405	417	450	93%
Good Health Products	Good Health	Omega 3 Fish Oil Health Guard 1000 mg			286		293	289	300	96%
Good Health Products	Good Health	Super Omega 3 Health Guard Fish Oil			524		497	511	500	102%
Health & Herbs International	Radiance	Omega 3 EPA & DHA			281		261	271	300	90%
Health World	Ethical Nutrients	Hi-Strength Fish Oil Capsules		779	768	732		760	694	109%
Integria Healthcare	Thompson’s	Fish Oil 1500	486		453	415		451	450	100%
Integria Healthcare	Thompson’s	Salmon Oil Plus 1000 mg			261		248	254	300	85%*
Lighthouse Supplements	Lighthouse	Salmon Oil 1000			274		253	263	300	88%*
Natural Health Laboratories	Clinicians	Omega-3 Fish Oil 1000 mg		290	288	269		282	300	94%
Natural Health Laboratories	Clinicians	Omega-3 Fish Oil 1500 mg High Strength	468		436	399		434	450	96%
Nordic Naturals	Nordic Naturals	Ultimate Omega		530	532	516		526	550	96%
PharmaCare Laboratories	Bioglan	Kids Smart Omega-3 Fish Oil	159		147	142		150	161	93%
PharmaCare Laboratories	Bioglan	Kids Smart Omega-3 Fish Oil Trio	179		150	138		156	161	97%
PharmaCare Laboratories	Bioglan	Super Fish Oil			265		274	269	275	98%
Real Vitamins	Sanderson	Fish Oil 2000		586	585	549		573	600	96%
Real Vitamins	Sanderson	Fish Oil 3000	953		868	817		879	900	98%
Red Seal Natural Health	Red Seal	High Potency Fish Oil 1,500 mg			419		416	417	450	93%
Sanofi Consumer Healthcare	MicroGenics	1500 mg Odourless Fish Oil		442	447	412		434	450	96%
Sanofi Consumer Healthcare	MicroGenics	Wild 1000 mg Fish Oil			288		263	275	300	92%
Sanofi Consumer Healthcare	Nature’s Own	Concentrated Fish Oil 1-A-Day Odourless			586			586	520	113%
Sanofi Consumer Healthcare	Nature’s Own	Fish Oil 1000 mg			291		263	277	300	92%
Sanofi Consumer Healthcare	Nature’s Own	Fish Oil 2000 mg			593		516	555	600	92%
Sanofi Consumer Healthcare	Nature’s Own	Odourless Fish Oil 1000 mg		301	302	272		292	300	97%
Sanofi Consumer Healthcare	Nature’s Own	Odourless Fish Oil 1500 mg		446	446	408		433	450	96%
Sanofi Consumer Healthcare	Nature’s Own	Odourless Fish Oil 2000 mg	595		581	531		569	600	95%
Swisse Wellness	Swisse	Ultiboost Odourless Wild Fish Oil	353		326	296		325	300	108%
Vitaco Health	Healtheries	Fish Oil 1000 mg			231		258	245	300	82%*
Vitaco Health	Healtheries	Fish Oil 1500 mg			432		423	427	450	95%
Vitaco Health	NutraLife	Fish Oil 1000 mg			280		284	282	300	94%
Vitaco Health	NutraLife	Fish Oil 1500 mg			430		407	418	450	93%
Vitaco Health	NutraLife	Fish Oil 1500 mg Plus Vitamin D			423		436	430	450	95%
Vitaco Health	NutraLife	OceanClean Triple Strength Omega 3	1,094		984	928		984	900	109%
Vitaco Health	NutraLife	Omega Smart Bites		456	466	451		458	423	108%

^*^Indicates products where the average result from all laboratories did not meet Australian regulations for nutrient content label claims (<90% of the labeled content).

### Comparison

A summary of the percentage of fish oil products that passed each measure in the tests carried out in the present study, in comparison to those reported by Albert *et al*. is provided in Table 5. A large difference existed in the proportion of fish oil products that comply with the maximum limit for PV (72% vs 17%, respectively) and in compliance with EPA/DHA label claim (91% vs 9%, resp.). A smaller difference was found for p-AV compliance, 64% vs 75%, respectively, if the p-AV limit is also applied to the flavored oils. Excluding flavored oils from the current study, the difference was 86% vs 75%.

**Table 5:** Comparison of values of oxidative quality and EPA/DHA content in finished products available on the New Zealand market, as reported by Albert and colleagues and the current study.

Parameter	Percentage of tested products that complied with the GOED quality criterion for oxidative status (PV max. 5 meq O_2_/kg; p-AV max. 20; TOTOX max. 26), and that adhered to labeled content of EPA plus DHA. (*only for unflavored fish oils)	
	Current study (n = 47)	Albert *et al*. (n = 36)
PV	72%	17%
p-AV	86%*	75%
TOTOX	77%*	50%
EPA + DHA content compliance to label claim	91%	9%

### Primary and secondary oxidation

**Figure 2:**
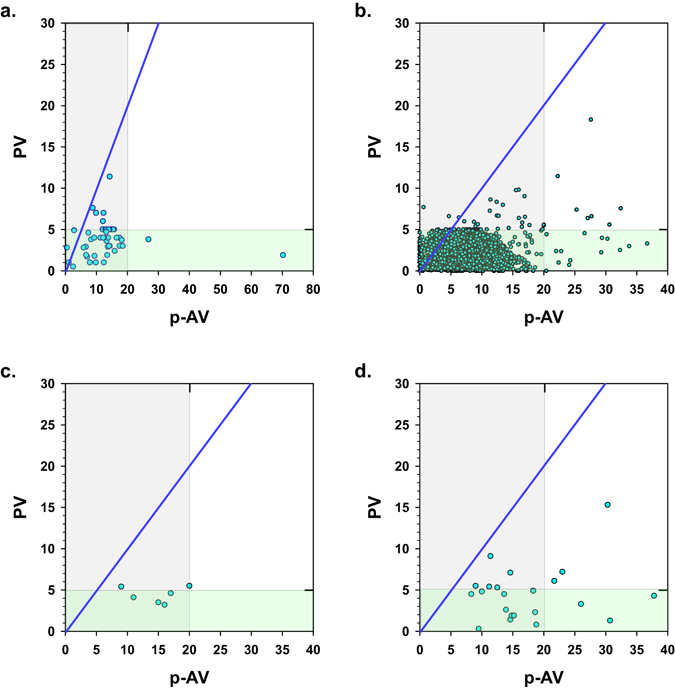
PV/p-AV ratios of fish oil products tested by third-party test organizations (panel a: LabDoor.com, panel b: IFOS; note, axis scales and symbols changed to improve visibility of data points) and reported in published literature (panel c: Turner *et al*.^33^, panel d: Madsen & Dyerberg, 1990^34^). The green- and grey-shaded areas fall below the GOED maximum PV and p-AV limits of 5 meq O_2_/kg and 20, respectively. The blue line represents a PV/p-AV ratio of 1.

Because a relative predominance of primary oxidation compared to secondary oxidation in marine oil products may provide an indication of peroxidation that has occurred shortly prior to analysis due to recent exposure to air, the relation between the PV and p-AV in fish oil products was determined for a number of public omega-3 LCPUFA oil testing reports and studies in which results for both parameters (PV and p-AV) have been published. To address this, the ratio of PV/p-AV^22^ was determined for each product tested and published by LabDoor.com ^30^, IFOS^32^, Turner *et al*.^33^, Madsen & Dyerberg^34^, the current study, and by Albert *et al*.^28^. All products analyzed in the current study were found to have PV/p-AV ratios <1, irrespective of the use of flavors (Fig. 1, panel a,b,c). In the study by Albert *et al*., 12 of the 32 tested fish oil products (38%) had PV/p-AV ratios >1 (Fig. 1d). The PV/p-AV ratios for marine oil products analyzed by LabDoor, IFOS, Madsen & Dyerberg, and Turner *et al*. were <1 for nearly all products (Fig. 2, panels a,b,c,d). In the IFOS analyses, three products that had been reported to be non-compliant with GOED limits (PV values > 5 meq O_2_/kg), out of more than 2000 tested marine oil samples, were found to have PV/p-AV ratios >1. All other products tested by IFOS with PV/p-AV ratios >1 had PV values below 5 meq O_2_/kg.

### Retesting oils close to expiry date

**Figure 3:**
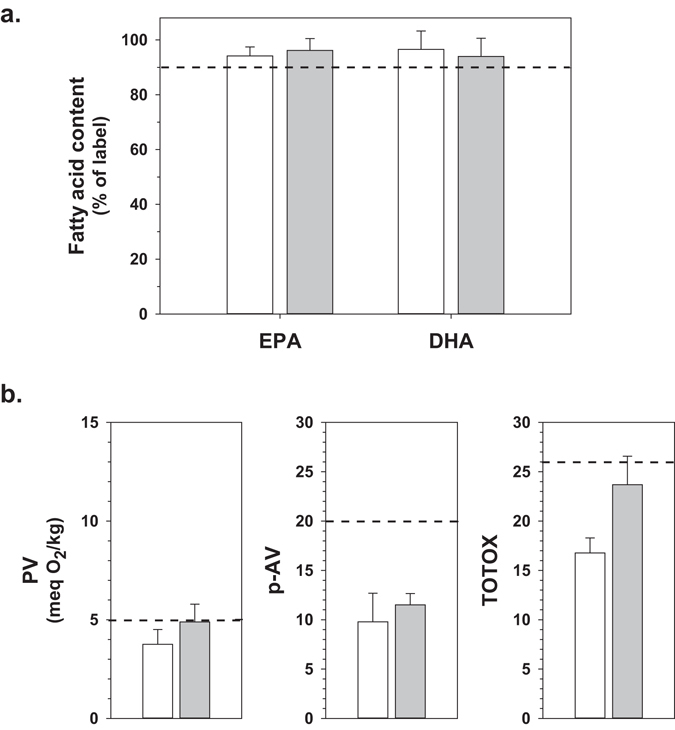
Content of EPA/DHA and oxidative status of several New Zealand fish oil products that were within one year before expiry date (white bars), and after retesting a year later after having passed expiry date (grey bars). Panel a: EPA and DHA content expressed as percentage of labeled content (means ± S.D., n = 5 products). The dashed line indicates the minimum required content of EPA and DHA according to local regulations. Panel b: PV (left), p-AV (middle) and TOTOX (right). Values are means ± S.D., n = 5 products for PV, and n = 3 for p-AV and TOTOX as two products were flavored and could not be tested for p-AV and TOTOX). The dashed lines indicate the maximum levels of PV, p-AV and TOTOX as specified by GOED.

Five of the tested fish oil products were within one year of shelf-life expiry, and were retested one year later (passed expiry date). The content of EPA and DHA did not significantly change in this period (Fig. 3a). PV, p-AV and TOTOX values increased, with PV and TOTOX values close to the limits of 5 meq O_2_/kg and 26, respectively (Fig. 3b).

## Discussion

The current study evaluated the oxidative status and EPA/DHA content of 47 fish oil dietary supplements sold on the New Zealand market. Of the tested fish oil products, 72% complied with the maximum permitted level of primary oxidation, 86% complied with secondary oxidation limits, and 91% with EPA/DHA content claims. The current study provides a high level of confidence in the accuracy of the obtained test results since multiple accredited laboratories tested the same samples, and obtained acceptable reproducibility in the results on oxidative status and omega-3 LCPUFA content. These results indicate that most, but clearly not all, of the analyzed fish oil products comply with standards for oxidative quality and EPA/DHA content voluntarily set by GOED. The current findings concur with results from third-party product quality monitors, such as Consumer Reports^29^, Labdoor.com ^30^, ConsumerLab.com ^31^, and the International Fish Oil Standards Program^32^, all of which report their test results publicly and note that a majority of fish oil supplements are compliant with oxidation limits and EPA/DHA label claims. No evidence for a high level of fish oil product non-compliance in New Zealand, as reported by Albert and colleagues^28^, could be found. On the contrary, a higher level of compliance is observed, but there are still products above the industry’s self-imposed limits of oxidation.

GOED standards are stricter than most pharmacopeial standards and global regulations, and maximum limits have been voluntarily set by producers and finished product manufacturers associated with GOED^5^. If the less stringent limits for pAV, PV and TOTOX used by the European and British Pharmacopeias and the Australian authorities would be held as reference in the current study, even higher rates of compliance can be concluded. For example, 100% of the tested fish oils complied with a p-AV limit of 30, 98% with a PV limit of 10 meq O_2_/kg, and 96% with a combined TOTOX value of 50. In other words, the reported level of product compliance in published studies depends on the regulatory framework used, but no evidence for a large-scale non-compliance of fish oil supplements in New Zealand is found with regard to oxidation or EPA/DHA content.

Since the products tested by Albert *et al*. were not disclosed (36 products), an exact comparison of the products tested in the present study (47 products) cannot be made. There is likely overlap in the product sets, because the products tested here cover all encapsulated fish oil products that were available on online pharmacies in the New Zealand market at the time of purchase. Informatively, 75% of fish oil products tested by Albert *et al*. were found to comply with secondary oxidation limits, similar to our findings, but a high incidence of non-compliance was found in the levels of peroxide (only 17% of fish oil were reported to be below 5 meq O_2_/kg). In order to better appreciate if high PV levels more frequently accompany otherwise compliant p-AV levels, the ratios of PV to p-AV were calculated for results from public testing records of several consumer organizations, as well as those reported in scholarly publications. It was found that marine oil products with a relatively elevated PV value compared to corresponding p-AV value (taken as a PV/p-AV ratio higher than 1) were not typical at all for the large majority of test results. In contrast, the reported proportion (38%) of fish oil supplements with a PV/p-AV ratio higher than 1 in Albert *et al*. is comparatively high. An elevation in PV is suggestive of recent oxidation having occurred in the fish oil samples, for example during a too lengthy sample preparation with exposure to ambient air, unadvised storage of oil samples after isolation of oil from gelatin capsules, handling with unsuitable laboratory materials that promote oxidation, *e.g*. plastic tubes or improperly washed glass tubes that are contaminated with transition metals, insufficient oxygen displacement in case of (unadvised) oil storage, and/or overexposure to light. It is unlikely in a retail environment for fish oil supplements to be exposed to oxygen, light or other catalysts of oxidation that would cause an acute elevation in recent oxidation since storage conditions are relatively stable.

In addition to avoiding inadvertent oxidation of samples of very easily oxidizable fish oil, a number of specific aspects regarding the analysis of oxidation in PUFA-rich oils are important. First of all, PV is expressed by weight, and not by volume. Adjustment of this aspect may lead to different rates of compliance in reports. Secondly, the validity of p-AV and TOTOX results is limited for many marine oil products that contain flavorings, because the assay for the p-AV measurement is susceptible to interference by a number of substances used for flavoring oils, generally leading to false positive overestimation of p-AV values, and incorrect attribution of non-compliance due to oxidation of PUFAs. Interference is flavor type- and concentration-dependent and should be evaluated for each individual product type. Twenty-five of the 47 tested products declared flavorings on their labels, several of which are empirically known to interfere with the measurement of p-AV. Restricting the analysis of p-AV to unflavored products confirmed that compliance with the p-AV limit was higher, and illustrated that a significant number of flavored oils on the market give false positive readings and cannot be reliably tested with current p-AV tests. In addition, we noted a higher inter-laboratory variance in p-AV results for flavored oils than for unflavored oils, suggesting multiple sources of interference, and further affirming the poor reliability of p-AV testing for flavored oils. It is of interest to note that several studies measuring secondary oxidation in flavored marine oil products have referenced the GOED Voluntary Monograph, a product quality standard that was created to ensure consumers can buy high quality products, but the GOED Voluntary Monograph excludes formulated products like flavored oils from its scope due to the interference with the p-AV tests. In other words, levels of non-compliance with secondary oxidation carry a risk for inflation if flavored omega-3 LCPUFA-rich oils are included in p-AV testing. This can also be appreciated in a recent report that focused on oxidation levels in North American omega-3 supplements^24^.

Of the 47 products tested for EPA/DHA content, only four products failed to meet their label claims when fatty acid content was expressed properly. This low non-compliance rate of 9% stands in stark contrast to the failure rate of 91% reported by Albert *et al*.^28^. The results of the current study lend support for the notion that an unacceptable level of inter-laboratory variability and errors in sample handling, analysis, and reporting could underlie the marked divergence in marine oil EPA/DHA content compliance that has been highlighted in recent reports^17–23, 28^. Possible cases of over-reporting of content may possibly also be noted in the literature^16, 35^. This suggests that laboratories meet significant and potentially overlooked technical challenges in making accurate fatty acid measurements in PUFA-rich oils in both directions.

It is critical that EPA/DHA content is expressed in absolute weights (not area percentages obtained from chromatograms), and also in the same chemical form if content is to be compared across products. For example, for all products labeled for sale in Australia, of which the majority of New Zealand fish oils in the present study were, EPA and DHA results should be expressed as triglycerides for the sake of accuracy. Ideally, content should be calculated as free fatty acid equivalents on a weight/weight basis, for example mg EPA per g oil product, and then expressed on the label according to applicable regulation. Compliance with EPA and DHA contents claimed on product labels should also be assessed against a relevant regulatory threshold. For example, in Australia and New Zealand the regulations indicate that a product should contain at least 90% of claimed content^36^, not 100% as asserted by several scholarly publications. In some countries this allows for some leniency in the natural variations in product compositions, as well as for a limited degree of variance in analytical measurements of fatty acid contents.

The oxidative deterioration of fish oil products has been suggested to contribute to the loss of EPA/DHA in a recent report^28^. Apart from the likelihood that the purported loss of EPA/DHA may be an artefactual finding, in the current study such a relationship could not be established, as illustrated by test results of five products that were reaching their expiry date and that were retested one year later, after shelf life expiry. Whereas indices of primary and secondary oxidation increased and were close to maximum limits, the EPA and DHA content was unaltered and stayed within label claims. This does not mean that high levels of oxidation cannot lead to losses of omega-3 content in marine oils, but at the relatively low levels of oxidation (and below industry limits) occurring in most fish oil products during their shelf life, oxidation is not of a sufficient magnitude to contribute to a net decrease in EPA/DHA content. Omega-3 levels below label claims, when they occur, are generally due to the (inappropriate) use of oils with inferior content of omega-3 fatty acids being used in formulation. A limitation in all studies, including the current study, is that batch-to-batch variation in oxidation has not been addressed. Batch-to-batch variation is however unlikely to explain the high level of non-compliance reported by Albert *et al*.^28^.

Under normal storage conditions, omega-3 finished products will generally very slowly oxidize yet stay within the acceptable and applicable limits of oxidative stability during shelf-life^13^. The results of the present study highlight potential issues in the execution of the methods that may have contributed to recent studies reporting high rates of fish oil product non-compliance. It is very important that producers of fish oils and finished product manufacturers set product specifications and claims with a reliable test method in mind, and use or select a laboratory with accreditation or demonstrated proficiency for analysis. The conclusion that fish oil products in New Zealand are “highly oxidized”, as qualified in the title of the report by Albert *et al*., appears overstated. Apart from the fact that most products in New Zealand are adhering to the tightest maximum limits, those products that would have exceeded these self-imposed industry limits are still meeting Australian, British Pharmacopeia, and European Pharmacopeia oxidation limits. To provide an added perspective, the limits on oxidation for marine oils are considerably more tight than those for vegetable and seed oils, which are much more commonly used as food ingredients and in household cooking and frying manipulations that induce much higher levels of PUFA oxidation, and the dietary intake of which is much larger than the typical doses of supplementary fish oils consumers may take. A recent study carried out on the quality of fish oils and omega-3 concentrates common to Australia and New Zealand reported that when accredited laboratories and standard protocols were used, the products clearly met their label claims for EPA and DHA content and were not oxidized^16^.

In summary, the evaluation of fish oil products available to consumers in New Zealand shows that a large proportion is compliant with EPA/DHA content and oxidative status. Up to 28% of fish oil supplements exceeded the GOED limit on PV, and 9% did not adhere to EPA/DHA label claims. This study also draws attention to the likelihood that the much higher rates of non-compliance reported by some recent studies in this area may be anomalous. Increased attention is invited to be paid to avoiding inadvertent oxidation of PUFA-rich oils during sample preparation, appreciation of interference in colorimetric assays, and improving analytical methodology and adequate reporting, to achieve improved quantitative assessments of the quality of PUFA-rich oils and products.

## Methods

### Tests

To perform as comprehensive a comparison as possible with the undisclosed fish oil supplements that had been tested in the study by Albert *et al*.^28^, every encapsulated fish oil supplement available at retail stores or internet retailers in New Zealand was purchased from internet retail sites based in New Zealand. Oils that were not encapsulated, or included omega-3s from other sources, were excluded. In total, 47 fish oil products were purchased. Once the products were received, they were stored sealed at room temperature up to seven weeks before sample distribution to participating analytical laboratories. Bottles were opened and the fish oil capsules were divided into numbered amber glass bottles, the numbers assigned to each sample logged, and then each sample product was tested at 2–3 validated and accredited laboratories that participate in inter-laboratory proficiency testing through the American Oil Chemists Society (AOCS)^11^. The analytical laboratories involved in the analyses were located at Alkemist Labs (Costa Mesa, CA, U.S.A.), DSM Nutritional Products (Columbia, MD, U.S.A.), Eurofins Central Analytical Laboratories (New Orleans, LA, U.S.A.), Omega Protein (Houston, TX, U.S.A.), and Nutrasource Diagnostics Inc. (Guelph, ON, Canada). The samples were blinded to ensure that the laboratories did not know the label declarations for EPA and DHA content. All products were tested by more than one laboratory for each parameter. In addition, all 47 products were tested by Eurofins Central Analytical Laboratories since this laboratory has been rated highly proficient at analyzing fish oils in the AOCS laboratory proficiency program multiple times since 2009.

All participating laboratories were directed to use one of the following methods for measuring fatty acid content: the European Pharmacopoeia method Ph.Eur. 2.4.29, the United States Pharmacopeia method USP 401 “Fats and Fixed Oils”, the official AOCS methods Ce 1i-07, the GOED analytical method “Assay for EPA and DHA”, or a quantitative method equivalent to the latter. For measuring PV and p-AV the AOCS methods Cd 8b-90 and Cd 18–90, respectively, were recommended, since these are widely used and accepted methods in the industry for the determination of primary and secondary oxidation products in oils. The total oxidation value of the oils was expressed as the TOTOX value, and calculated as follows: (2 x PV) + p-AV. Information on the analytical methodologies and quality controls used by each participating laboratory for fatty acids analysis, PV and p-AV, are provided in the Supplementary Materials section.

Flavored fish oils received an additional separate analysis due to the known interference of flavors with p-AV testing, although no alternate methods are specified in any monograph, pharmacopeia or regulations for oils containing flavors. All of the flavored oils were tested at Nutrasource Diagnostics Inc using a proprietary True Anisidine Value (TAV) assay methodology, a HPLC-UV-based method developed to test secondary oxidation products in flavored fish oils. Oils with a value over 70% are considered to meet the criterion for having a low level of secondary oxidation.

For the purpose of this study, a PV ≥ 5, a p-AV ≥ 20, and a TOTOX value ≥ 26 were considered to be unacceptable for unflavored samples, while a PV ≥ 5 was considered unacceptable for flavored oils. A natural fish oil capsule is required to contain a minimum of 90.0% of the label claim of EPA and DHA, and applies to the components that are quantified on the label of a multicomponent active ingredient of natural origin when the proportions of these components vary independently of each other. This specific requirement follows the Australian regulations for active ingredients in therapeutic goods, which the dietary supplement market in New Zealand uses as its regulatory framework in the absence of local regulation^36^. A limited number of the fish oil products that were nearing the end of shelf-life, *i.e*. within one year of their expiry date, were retested one year later. Between the first analysis and retesting, fish oil products were stored under shelf-life conditions at room temperature. Published testing reports of the oxidative status (PV, p-AV, TOTOX) of omega-3 LCPUFA supplements were obtained from the websites of LabDoor.com ^30^, the International Fish Oil Standards program (IFOS)^32^, as well as from published results in Turner *et al*.^33^, Madsen & Dyerberg^34^, and Albert *et al*.^28^.

## Electronic supplementary material

Supplementary Information on Methods
