# Supplementary material for: Omega-3 Long-Chain Polyunsaturated Fatty Acid Content and Oxidation State of Fish Oil Supplements in New Zealand
Source: Sci Rep. 2017 May 3;7:1488. doi: 10.1038/s41598-017-01470-4 (PMC5431122; doi:10.1038/s41598-017-01470-4)
Supplement: Supplementary file 1 — Supplementary Information on Methods [file 41598_2017_1470_MOESM1_ESM.pdf]

SUPPLEMENTARY INFORMATION ON METHODS

Title: OMEGA-3 LONG-CHAIN POLYUNSATURATED FATTY ACID CONTENT AND OXIDATION STATE OF FISH OIL SUPPLEMENTS IN NEW ZEALAND.

Authors: Gerard Bannenberg, Craig Mallon, Holly Edwards, Derek Yeadon, Kevin Yan, Holly Johnson & Adam Ismail.

Supplementary information on testing by participating laboratories:

Eurofins:

Fatty acids (EPA/DHA):

Reference Method: AOCS Ce 1-89

Applicability: Marine oils and marine oil esters in area % and mg/g EPA and DHA.

Description: Internal standard is added to sample; sample is heated and reacted under nitrogen with NaOH. BF_3 is added and sample is heated and reacted under nitrogen. Hexane is added and sample is vortexed under nitrogen. NaCl solution is added, sample is thoroughly mixed, and a portion of the hexane layer is used for analysis by GC-FID.

Number of replicates: Minimum 2 per sample unless otherwise specified

Precautions taken: Samples stored refrigerated (~4°C) prior to analysis (except for capsules which are stored at room temperature); samples kept under nitrogen during sample preparation.

Accuracy: ≤2% for mg/g results, ≤1% for area % results.

Specificity: Analysis delineates EPA and DHA peaks by comparison to multiple standards and/or standard mixes.

Sensitivity: LOQ is 1.0 mg/g.

Blanks used: 2 or more solvent blanks are analyzed with each analytical batch.

Inter-day precision: ≤2% for mg/g results, ≤1% for area % results.

Total within-laboratory deviation: ≤2% for mg/g results, ≤1% for area % results.

Primary reference standards: Sigma-Aldrich FAME 37 mix, DHA and EPA neat standards from Nu-Chek Prep, internal laboratory control samples analyzed with each batch.

Laboratory accreditations: AOCS Marine Oil FAP, AOCS GOED series.

Peroxide Value:

Reference Method: AOCS Cd 8-53

Applicability: All normal animal and vegetable fats and oils.

Description: Sample is dissolved in 3:2 acetic acid:chloroform solution and KI is added. Solution is mixed, and allowed to sit for 1 minute. DI water is added, and the sample is

titrated with sodium thiosulfate solution. SDS and starch indicator are added, and solution is titrated to a clear endpoint.

Number of replicates: Minimum 1 per sample, at least 1 sample per batch analyzed in duplicate.

Precautions taken: Samples stored unopened until Peroxide Value and p-Anisidine Value (if requested) are run. Samples stored at either room temperature (~20°C) or refrigerated; capsules are stored at room temperature, loose oils are stored refrigerated (~4°C).

Accuracy: 5%.

Specificity: Detects substances that oxidize KI under the conditions of the test; these substances are generally assumed to be peroxides or other similar products of fat oxidation.

Sensitivity: LOQ is 0.10 mEq/kg.

Blanks used: A blank is analyzed with every analytical batch. (One flask of 3:2 solution is analyzed without addition of sample).

Inter-day precision: 5%.

Total within-laboratory deviation: 5%.

Primary reference standards: Internal laboratory LCS.

Laboratory accreditations: AOCS Marine Oil series, AOCS GOED series.

p-Anisidine Value:

Reference Method: AOCS Cd 18-90.

Applicability: All normal animal and vegetable fats and oils.

Description: Sample is filtered if necessary to remove water. Sample is dissolved in a known volume of isooctane, and the absorbance at 350 nm is measured using a spectrophotometer. A portion of the sample is reacted with p-anisidine reagent, and after 10 minutes the absorbance at 350 nm of the reacted solution is measured.

Number of replicates: 2 minimum per sample.

Precautions taken: Samples stored unopened until Peroxide Value and p-Anisidine Value tests are run.

Accuracy: 5%.

Specificity: Detection is specific to aldehydes (formed from secondary lipid peroxidation); solutions containing aldehydes (particularly flavoring compounds such as vanillin, benzaldehyde, etc.) will give artificially higher results.

Sensitivity: LOQ is 1.0 (value is unitless).

Blanks used: Isooctane is used as a blank with each sample after reaction with p-anisidine solution.

Inter-day precision: 5%.

Total within-laboratory deviation: 5%.

Primary reference standards: Internal laboratory LCS.

Laboratory accreditations: AOCS Marine Oil series, AOCS GOED series.

DSM:

Fatty acids (EPA/DHA):

The fish oil samples were analysed for the content of EPA and DHA after conversion into fatty acid methyl esters, and analysed using an in-house method equivalent to the GOED Fatty Acid method and AOCS Ce 1-62, 'Fatty Acid Composition by Gas Chromatography', with modified chromatographic conditions. Oil samples were analysed immediately after being extracted from the product capsules. After each step, following addition of each reagent, the test tube headspace was purged with nitrogen and the tube was sealed using a screw-cap with an inert liner. A portion of the sample oil (approx. 25mg) was accurately weighed into a glass screw-cap test tube. The oil was dissolved in 1mL of a toluene solution containing an internal standard (methyl nonadecanoate) and 50ppm butylated hydroxytoluene. After purging with nitrogen, the samples were saponified by reaction with 4mL 0.5N methanolic sodium hydroxide at 110°C for 5 minutes. The resultant free fatty acids were then esterified by reaction with 5mL 14% methanolic boron trifluoride for 30 minutes at 110°C. Methyl esters were extracted with hexane, and an aliquot was transferred to a GC vial for analysis. Methyl esters (1 µl injection volume) were separated by gas chromatography (Agilent Technologies 7890 gas chromatograph) using a column coated with nitroterephthalic-acid-modified polyethylene glycol (15 m x 0.1mm x 0.1 um film thickness; Agilent 127-32H2). Hydrogen was used as carrier gas at constant pressure (50 psi). The oven temperature was held at 150°C for 0.25 min, raised at 20°C min^{-1} to 200°C and held for 1 min, then increased at 5°C min^{-1} to 225°C and held for 2 min, and thereafter

raised to 245°C at 80°C min^{-1} and held for 1 min. Fast run times of approximately 12 min were achieved. The injector (split mode 1:200) and flame ionization detector (40 ml min^{-1} hydrogen) were kept at 250°C. Nitrogen was used as make-up gas (25 ml min^{-1}) to improve sensitivity.

As a quality control, an oil standard is included with each data set that was previously characterized as part of an interlaboratory study. Quality control results were within the 95% confidence interval defined by the interlaboratory study. DHA and EPA (as their methyl esters) were identified by comparison of their retention times to those of methyl DHA and EPA standards (Nu Chek Prep., Elysian, MN, USA; >99% purity). DHA and EPA were baseline resolved. Reagent blanks, in which the procedure was followed with the sample oil omitted, were run to verify that there were no false positives or interferences from the reagents, glassware or system.

A solution containing methyl DHA, methyl EPA, and internal standard (methyl C19:0) was analyzed to determine relative response factors (RRF) for DHA and for EPA:

$$RRF\ (X) = \frac{Area_{ISTD}}{Area_X} \times \frac{Weight_X}{Weight_{ISTD}}$$

$Area_{ISTD}$ = area of methyl C19:0 peak from Rf injection

$Area_X$ = area of methyl DHA or methyl EPA from Rf injection

$Weight_{ISTD}$ = weight of methyl C19:0 in Rf solution

$Weight_X$ = weight of methyl DHA or methyl EPA in Rf solution

DHA and EPA contents were calculated on a free fatty acid (FFA) basis as follows:

$$Potency\ (X)(mg\ per\ gram) = \frac{area_X}{area_{ISTD}} \times \frac{Weight_{ISTD}}{Weight_X} \times RRF\ (X) \times \frac{MW_{FFA}}{MW_{ME}} \times 1{,}000$$

$Area_X$ = peak area of methyl DHA or methyl EPA

$Area_{ISTD}$ = area of internal standard peak

$Weight_{ISTD}$ = weight of internal standard in sample preparation (mg)

$Weight_X$ = sample weight (mg)

RRF (X) = response factor of DHA or EPA relative to internal standard

MW_{FFA} = molecular weight of DHA or EPA in free fatty acid form

MW_{ME} = molecular weight of DHA or EPA in methyl ester form

In order to compare results to the product label claims, DHA and EPA contents were converted to a triglyceride (TAG) basis as follows:

$$mg\ triglyceride\ per\ gram = Potency\ (X) \times \frac{MW_{TAG}}{3 \times MW_{FFA}}$$

MW_{FFA} = molecular weight of DHA or EPA in free fatty acid form

MW_{ME} = molecular weight of DHA or EPA in triglyceride form

Samples were analysed in duplicate. Individual and averaged results were reported. Relative standard deviation (RSD) between replicate analyses ranged from 0.1 – 4.8%, with the majority having an RSD <2.0%.

Peroxide Value:

Testing was performed according to AOCS official method Cd 8-53. The contents of at least ten capsules were pooled to obtain a representative sample. Immediately prior to analysis, the capsules were punctured and their oil contents pooled into a glass vial, whose headspace was purged and blanketed with argon until a sample was withdrawn for analysis. All analysis was performed within 30 minutes of puncturing the capsules. 5.0 g of oil was accurately weighed into a titration beaker and dissolved in 30mL of 3:2 glacial acetic acid / chloroform solution. 500µl of saturated potassium iodide solution was added. The solution was mixed on a magnetic stir plate, then allowed to sit with occasional shaking for exactly 60 seconds, after which 30 ml of distilled water was added. The solution was titrated with 0.01 N sodium thiosulphate solution, with the endpoint determined using an autotitrator. Peroxide value was calculated as milliequivalents of peroxide per kilogram of oil sample. Analysis was performed in singular, on a sample obtained by pooling the contents of ten capsules. Two previously characterized omega-3 oils are run as quality control checks (one low and one high), and came within their expected ranges of 0.55 – 0.60 and 3.90 – 4.05 meq/kg, respectively.

p-Anisidine Value:

Testing was performed according to AOCS Cd 18-90. This is a widely used and accepted method in the industry for the determination of secondary oxidation products in oils. As previously noted, it is known that certain flavorings may interfere for the analysis and lead to false positives. Since the samples were submitted blind, the analysis was performed as described, unknowing to the analyst which samples may contain such flavorings. The contents of at least ten capsules were pooled to create a representative sample. Immediately prior to analysis, at least ten capsules were punctured and their oil contents pooled into a glass vial, whose headspace was purged and blanketed with argon until a sample was withdrawn for analysis. The oil was immediately analyzed within 30 minutes of puncturing the capsules, according to the following procedure (based on AOCS method Cd 18-90), used to determine aldehydes as a measure of secondary oxidation. A portion of the oil sample (0.25g) was accurately weighed, and dissolved in 25mL isooctane. Any sample solutions that appeared turbid were passed through a 0.45µm PTFE syringe filter. The initial absorbance of this solution was then measured at 350nm in a 1cm path length cuvette using a UV-vis. spectrophotometer. Isooctane was used as a blank. 5mL of the sample solution was transferred to a test tube. 1mL of para-anisidine reagent (0.25g/100mL in glacial acetic acid) was added, and the tube was capped and shaken. After exactly 10 min., the absorbance of this solution was measured at 350nm, against the absorbance of a reagent blank.

The p-Anisidine Value of the sample was determined as follows:

$$p - A.V. = \frac{25 \times (1.2\, As - Ab)}{m}$$

Where:

As = absorbance of sample solution after the ten-minute reaction with *p*-anisidine reagent

Ab = absorbance of initial sample solution

m = mass of oil in sample

Analysis was performed in singular, on a sample obtained by pooling the contents of ten capsules. A previously characterized omega-3 oil with known *p*-Anisidine Value was analyzed as a quality control, and came within the established range of 17 – 20 AV. Prior to analysis, the system suitability of the spectrophotometer was verified by measuring the wavelength and absorbance of a series of potassium dichromate solutions.

Omega Protein:

Precautions taken during sample storage and sample preparation: Nitrogen blanket of the samples, samples refrigerated when not being analyzed.

Fatty acids (EPA/DHA):

AOCS Ce 1i-07. All samples were converted to methyl ester derivatives regardless of whether they were ethyl ester (EE) or triglyceride (TG) oils as described in AOCS Official Method Ce 2-66. Triacylglycerol (23:0 TAG) (NuCheck Prep) was included as an internal standard prior to methylation for determination of the concentration of the individual FAME in the oil samples after methylation. The prepared FAME was dissolved in hexane and run on a gas chromatograph equipped with auto sampler, electronic pressure control, and flame ionization detector, using a capillary column type: ZB-WaxPlus 30m x 250 µm x

0.25 µm. Hydrogen served as the carrier gas at a constant flow rate of 1.2ml/min. The oven was programmed at an initial temperature of 170°C with a ramp of 1°C/min until a final temperature of 225°C was achieved. The injector port was set at a constant temperature of 235°C and the detector at 325°C. Number of replicates analyzed per sample: 3 replicates of each sample. Blanks used: Hexane was run every 4-5 samples to ensure no column contamination had occurred. Primary reference standards: GLC 714 (NuCheck Prep) was used for calibration of the instrument, GLC 674 (NuCheck Prep) was used for peak naming and identification, and also Marine PUFA Omega-3 (Sigma Aldrich) for peak naming and identification.

Peroxide Value: AOCS Cd 8b-90. Number of replicates analyzed per sample: 3 replicates of each sample. Primary reference standard: AOCS marine oil standard for laboratory proficiency program.

p-Anisidine Value:: AOCS Cd 18-90. Number of replicates analyzed per sample: 3 replicates of each sample. For p-AV, a blank was used as described in the AOCS method. Primary reference standard: AOCS marine oil standard for laboratory proficiency program.

Laboratory accreditations: AOCS Approved Chemist Marine Oils Fatty Acid Profile, AOCS Approved Chemist Marine Oil.

Alkemist Labs:

Fatty acids (EPA/DHA):

Method: GOED Voluntary Monograph v.3 – Assay (EPA and DHA)

The method is applicable for fish, plant, or microbial sources in bulk oils and encapsulated oil, either as triglycerides or as ethyl esters.

Short description of instrument/method set-up:

GC Instrument: Agilent 7890B, Column: CP-Wax 52 CB, 25m x 0.25mm x 0.2µm. Carrier gas: Hydrogen. Split ratio: 200:1, Injection volume 1 µL, Injector Temp: 250 °C, Detector temp: 270 °C

Sample Preparation:

Transferred approximately 400 mg to 10 mL low-actinic volumetric flask, and added approximately 70 mg methyl tricosanoate and filled to volume with 0.05 g/L BHT in TMP. Vortexed for 30 seconds. Transferred 2.0 mL to a low-actinic quartz vial. Evaporated under nitrogen at 40-50°C. Added 1.5 mL 20 g/L sodium hydroxide in methanol, covered with nitrogen and closed cap. Heated on steaming water bath at 90ºC for 7 minutes. Cooled to 40-50°C. Added 2.0 mL boron trichloride in methanol, displaced air with nitrogen and closed cap. Heated on steaming water bath at 90°C for 30 minutes. Cooled to 40-50°C and added 1.0 mL trimethylpentane. Closed cap and shook vigorously for 30 seconds. Immediately added 5 mL saturated sodium chloride, displaced air with nitrogen, closed cap and shook vigorously for 15 seconds. Transferred upper layer to separate low-actinic quartz tube. Added 1.0 mL trimethylpentane to the methanolic layer, displaced air with nitrogen,

closed cap and shook vigorously for 15 seconds. Transferred upper layer to the same secondary tube. Added 1.0 mL water, shook for 30 seconds and allowed layers to separate. Added another 1.0 mL water and shook for 30 seconds and added 100 mg anhydrous sodium sulfate. Allowed layers to separate and transferred top layer to vial for analysis.

Number of replicates analyzed per sample: 2

Precautions taken during sample storage and sample preparation: Low actinic glassware used. Nitrogen cover.

Blanks used: 0.05 g/L BHT

Primary reference standards:

Docosahexaenoic Acid Ethyl Ester, USP1224620, Lot GOK228, 99.1%

Eicosapentaenoic Acid Ethyl Ester, USP1234307, LOT HOL521, 99.1%

Methyl Palmitate, Sigma P5177, Lot MKBR6279V, exp. 06/24/17, 99.0 %

Methyl Stearate, Sigma 55376, Lot BCBKG253V, exp. 02/28/16, 99.8%

Methyl Arachidate, Sigma 10941, Lot BCBK7350V, exp. 02/28/16, 99.5%

Methyl Behenate, Sigma B3271, Lot MKBQ7490V, exp. 06/24/17, 99.3%

15-Tetracosenoic Acid Methyl Ester, Sigma 17265, exp. 12/31/16, Lot BCBM4670V, 99.9%

Laboratory accreditations: ISO 17025, AOAC Analytical Laboratory Accreditation Criteria Committee (ALACC), NIST proficiency for Fish Oils Exercise L 2016.

Nutrasource Diagnostics:

Fatty acids (EPA/DHA):

Method: Modified AOCS CE 1b-89. Applicable to triglyceride and ethyl ester oils.

Gas Chromatograph System (Varian 3900) with FID Detector. Gases ultra-high purity 5.0: Hydrogen, Air, Nitrogen, Helium. Gases, regular purity: Nitrogen.

Materials:

Methyl tricosanoate standard, Nu Chek Prep, Inc.; GLC-714 Reference Standard, Nu Chek Prep, Inc.; Supelco 37 component FAME (fatty acid methyl ester) Mix, Sigma; PUFA No. 1 (Marine Source), analytical standard, Sigma; Sodium hydroxide, reagent grade, Sigma or equivalent; Methanol, reagent grade, Caledon or equivalent; Boron trichloride in methanol (BCl_3-MeOH), 12 % solution, Sigma or equivalent; Butylated hydroxytoluene (BHT), Sigma or equivalent; 2,2,4-Trimethylpentane (iso-octane), reagent grade, Caledon or equivalent; Sodium chloride, reagent grade, Sigma or equivalent; Anhydrous sodium sulfate, reagent grade, Sigma or equivalent, Purified water, USP grade. Analytical balance, capable of accurately weighing to 0.01 mg. Test Tubes: 9 mL and 15 mL with PTFE-lined caps. Vials: 2 mL with PTFE-lined no-slit caps. Hot plate, vortex.

Procedural highlights:

Gas chromatography parameters:

Column: DB-WAX, 30 m x 0.25 mm ID x 0.15 μm film. Carrier gas: helium, constant flow 1.0 mL/min. Make-up gas: helium, 29 mL/min. Injector temperature: 250°C. Injection split ratio: 100. Injection volume: 1 μL. Detector temperature: 270 °C. Hydrogen flow: 30 mL/min. Air flow: 300 mL/min. Column Temperature Program: Stabilization time: 0.30 min (at least), Initial temperature: 170 °C, no hold time, Ramp: 1.8 °C/minute to 205 °C, no hold. Ramp: 1.4 °C/minute to 225, hold 1.27 minutes. Run time: 35 minutes, Next Injection Delay: 3 minutes.

Number of replicates analyzed per sample: 1

Precautions taken during sample storage and sample preparation: Nitrogen cover, store at room temperature, protected from light.

Primary reference standards: see method.

Peroxide Value: AOCS Cd 8b-90

p-Anisidine Value: AOCS Cd 18-90

TAV Test: In-House method, DTM-158-R00

Laboratory accreditations: AOCS LPP, Health Canada Establishment License.
